# Mainstream germline genetic testing in men with metastatic prostate cancer: design and protocol for a multicenter observational study

**DOI:** 10.1186/s12885-022-10429-2

**Published:** 2022-12-30

**Authors:** Michiel Vlaming, Eveline M. A. Bleiker, Inge M. van Oort, Lambertus A. L. M. Kiemeney, Margreet G. E. M. Ausems

**Affiliations:** 1 Division Laboratories, Pharmacy and Biomedical Genetics, dept. of Genetics, University Medical Center Utrecht, Heidelberglaan 100, 3584 CX Utrecht, The Netherlands; 2 Division of Psychosocial Research and Epidemiology, The Netherlands Cancer Institute, Plesmanlaan 121, 1066 CX Amsterdam, The Netherlands; 3 Department of Clinical Genetics, Leiden University Medical Center, Albinusdreef 2, 2333 ZA Leiden, The Netherlands; 4 Family Cancer Clinic, The Netherlands Cancer Institute, Plesmanlaan 121, 1066 CX Amsterdam, The Netherlands; 5 Department of Urology, Radboud university medical center, Geert Grooteplein Zuid 10, 6525 GA Nijmegen, The Netherlands; 6 Department for Health Evidence, Radboud university medical center, Geert Grooteplein Zuid 21, 6525 EZ Nijmegen, The Netherlands

**Keywords:** Prostate cancer, Mainstream genetic testing, Genetic counseling, Germline genetic testing

## Abstract

**Background:**

In international guidelines, germline genetic testing is recommended for patients with metastatic prostate cancer. Before undergoing germline genetic testing, these patients should receive pre-test counseling. In the standard genetic care pathway, pre-test counseling is provided by a healthcare professional of a genetics department. Because the number of patients with metastatic prostate cancer is large, the capacity in the genetics departments might be insufficient. Therefore, we aim to implement so-called mainstream genetic testing in the Netherlands for patients with metastatic prostate cancer. In a mainstream genetic testing pathway, non-genetic healthcare professionals discuss and order germline genetic testing. In our DISCOVER study, we will assess the experiences among patients and non-genetic healthcare professionals with this new pathway.

**Methods:**

A multicenter prospective observational cohort study will be conducted in 15 hospitals, in different regions of the Netherlands. We developed an online training module on genetics in prostate cancer and the counseling of patients. After completion of this module, non-genetic healthcare professionals will provide pre-test counseling and order germline genetic testing in metastatic prostate cancer patients. Both non-genetic healthcare professionals and patients receive three questionnaires. We will determine the experience with mainstream genetic testing, based on satisfaction and acceptability. Patients with a pathogenic germline variant will also be interviewed. We will determine the efficacy of the mainstreaming pathway, based on time investment for non-genetic healthcare professionals and the prevalence of pathogenic germline variants.

**Discussion:**

This study is intended to be one of the largest studies on mainstream genetic testing in prostate cancer. The results of this study can improve the mainstream genetic testing pathway in patients with prostate cancer.

**Trial registration:**

The study is registered in the WHO’s International Clinical Trials Registry Platform (ICTRP) under number NL9617.

## Background

Prostate cancer is the second most common cancer in men worldwide [1]. Most patients present with local prostate cancer, whereas at least 7% present with metastatic prostate cancer (mPC) [2, 3]. According to different studies, between 3 and 19% of men with mPC harbor a pathogenic germline variant in a cancer predisposition gene [4–18]. Pathogenic germline variants are hereditary and therefore family members can have these variants too. These variants are mostly prevalent in breast cancer genes *BRCA2*, *CHEK2* and *ATM* [4, 6, 7, 10, 12, 16, 18]. Depending on the affected gene, female family members who carry a pathogenic germline variant in one of these genes have a moderate to high risk of developing breast cancer and a moderate risk of developing ovarian cancer [19–21]. If female family members carry a pathogenic germline variant, they can decide to undergo preventive actions, e.g., periodic breast cancer screening, risk-reducing mastectomy or risk-reducing salpingo-oophorectomy [22–24]. Male family members with a pathogenic germline variant in the *BRCA2* gene have an increased risk of developing prostate cancer, for which periodic PSA screening is advised [25]. In addition, they also have an increased breast cancer risk [26].

Results of genetic testing can also be important for the treatment of the patient, since metastatic castration-resistant prostate cancer (mCRPC) patients with a pathogenic *BRCA* variant were shown to have longer progression-free survival when treated with poly (ADP-ribose) polymerase (PARP) inhibitors [27]. Therefore, PARP inhibitors are approved by the FDA and EMA for these patients with a pathogenic *BRCA* variant [28, 29].

Because of the possible consequences for family members and the extra treatment option when a pathogenic germline variant in one of the *BRCA* genes is found, germline genetic testing is currently recommended for patients with mPC in multiple international guidelines, e.g., NCCN, EAU and AUA guideline [30–32].

In the standard genetic counseling pathway, patients receive pre-test and post-test genetic counseling by a clinical geneticist or genetic counselor at a genetics department. Due to the large number of patients with mPC, this standard pathway might exceed the capacity in the genetics departments. Therefore, it is desirable to explore other ways of providing genetic counseling and testing, e.g., ‘mainstream genetic testing’. Mainstream genetic testing was first described by George et al. in 2016 [33]. In the mainstreaming pathway, pre-test counseling is performed by non-genetic healthcare professionals instead of a geneticist or genetic counselor. Mainstream genetic testing has mainly been implemented in patients with ovarian cancer and breast cancer [33–45], and on a small scale in prostate cancer [46, 47]. In ovarian cancer, non-genetic healthcare professionals were highly confident providing pre-test counseling and asking consent of patients and the large majority were willing to continue performing pre-test counseling in everyday practice [34, 40, 45]. Patients were highly satisfied with the counseling and the mainstreaming pathway [33, 34, 40, 45]. In two prostate cancer studies conducted in the US and Australia, both non-genetic healthcare professionals and patients considered mainstream genetic testing acceptable [46, 47]. Non-genetic healthcare professionals considered mainstream genetic testing as an efficient use of their time [47]; patients were satisfied and showed little depression or anxiety after pre-test counseling by an oncologist [46, 47].

## Objectives

We aim to implement a mainstreaming pathway for mPC. The primary objective of our DISCOVER (*Detecting Increased Susceptibility for Cancer in relatives by Offering genetic Variant Evaluation as Regular health care*) study is to explore on a larger scale non-genetic healthcare professionals’, such as urologists and oncologists, and patients’ experiences with mainstream genetic testing. We will assess their satisfaction with and acceptability of mainstream genetic testing, and the psychosocial impact of germline genetic testing in patients. We will assess their experience, including the knowledge of non-genetic healthcare professionals and patients, and we will assess whether patients inform their relatives about genetic testing when a pathogenic germline variant is identified. The secondary objective is to assess the efficacy of the mainstreaming pathway, based on the diagnostic yield of genetic testing and the time investment for non-genetic healthcare professionals.

## Educational support for non-genetic healthcare professionals

We developed an online training module to prepare non-genetic healthcare professionals to perform pre-test counseling and discuss and order genetic testing in patients with mPC.

### Assessment of needs and preferences of non-genetic healthcare professionals

To determine the content of the online training module, we first conducted two online focus groups and two interviews with non-genetic healthcare professionals who are involved in prostate cancer care. The goal of the focus groups and interviews was to assess the barriers and facilitators for mainstream genetic testing, to identify knowledge gaps and to determine their opinion about assessing a family history. Urologists (*n* = 6) from academic and non-academic hospitals participated in the first focus group. They were recruited via the Dutch working group on Oncological Urology and via personal invitations. The oncologists (*n* = 2), nurse specialists (*n* = 3) and a urologist who participated in the second focus group were recruited via personal invitations. Interviews were conducted with one nurse specialist and one urologist who could not attend the focus group, but were willing to participate.

The most important barrier for mainstream genetic testing in prostate cancer was a lack of knowledge about genetic testing, which was mainly reported by urologists. They were unaware of the medical and psychosocial consequences of a pathogenic germline variant in the patient and his (female) family members. They also did not see the benefits of germline genetic testing in elderly patients and patients without living family members. The main facilitator for mainstream genetic testing was a Frequently Asked Questions form for non-genetic healthcare professionals. Participating non-genetic healthcare professionals stated that they regularly asked whether patients have a positive family history for prostate and breast cancer. However, they were not used to asking about other *BRCA*-related cancers, e.g., ovarian and pancreatic cancer or about an Ashkenazi Jewish ancestry, which is associated with a higher prevalence of *BRCA* founder variants.

### Assessment of needs and preferences of patients

We conducted one online focus group and three interviews with prostate cancer patients to determine reasons to accept or decline germline genetic testing. An invitation to participate in the online focus group was disseminated by the Dutch prostate cancer patient organization and by the Dutch Uro-Oncology Study Group. In total, 39 patients wanted to participate and 8 patients were selected. We selected patients of varying age (from 58 up to 81 years) and tumor stage (from localized up to metastatic castration-resistant prostate cancer). Some patients already had personal experience with germline genetic testing.

Patients who had personal experience with germline genetic testing indicated that the main reason to undergo germline genetic testing was to find out if they had a pathogenic germline variant and thus if their family members had a significantly higher risk of developing cancer. This was also the main reason for patients without personal experience. Patients without personal experience with germline genetic testing however mainly focused on the risk of developing prostate cancer for male family members, while patients who had received genetic counseling and a germline genetic testing result also pointed out the risk for female family members of developing breast and ovarian cancer. The patients hypothesized that the most important reasons to decline germline genetic testing would be fear about the test result or fear about distress in the family.

### Development of an online training module for non-genetic healthcare professionals

After all focus groups and interviews were conducted, we developed an online training module for non-genetic healthcare professionals, who are treating prostate cancer patients. Online training modules that were previously developed at our department for gynecologists [48] and surgeons were used as a template and adapted based on the needs and preferences of non-genetic healthcare professionals and patients with prostate cancer. The framework of this training has similarities with multiple other training procedures in mainstreaming initiatives [49]. The online training module can be completed within 1 hour and consists of one introduction video and four knowledge clips. The content of the knowledge clips is shown in Table 1. The online training is accredited for urologists, oncologists and nurse specialists.

**Table 1:** Content of knowledge clips in the online training module

Knowledge clip	Content
1	• difference between germline and somatic pathogenic variants • difference between genetic testing in blood and in tumor tissue • role of pathogenic variants in carcinogenesis
2	• indications for germline genetic testing in prostate cancer • risks of developing cancer for male and female family members with a pathogenic germline variant • preventive measures for breast/ovarian cancer for female family members • PSA screening for male family members carrying a pathogenic germline variant in BRCA2
3	• protocol for mainstream genetic testing
4	• advice about discussing germline genetic testing with patients • a simulated conversation between a urologist and a patient • an interview with a prostate cancer patient who carries a pathogenic germline variant

## Methods

**Fig. 1:**
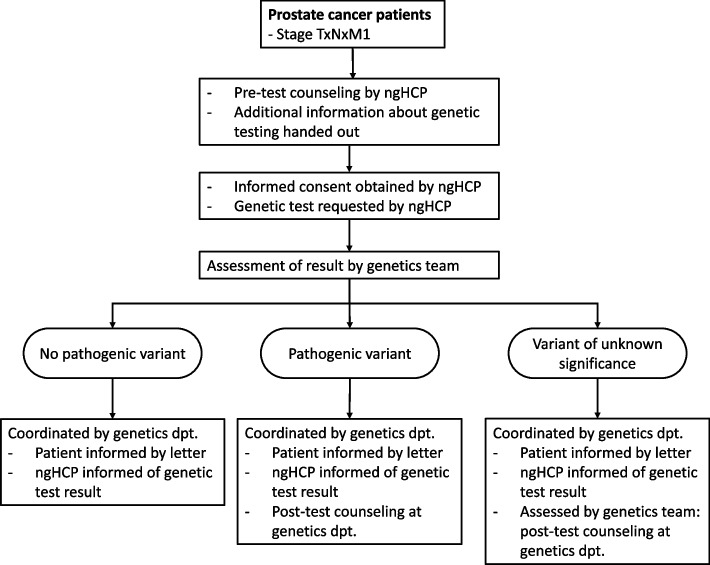
Mainstreaming pathway for patients with metastatic prostate cancer in the Netherlands. ngHCP: non-genetic healthcare professional, dpt.: department

This study is a prospective, observational cohort study that will be conducted in 15 hospitals in the Netherlands. In these 15 hospitals, a mainstreaming pathway will be implemented in daily practice, which is adapted from the pathway previously developed and implemented for genetic testing in patients with ovarian cancer [48]. In this pathway (Fig. 1), non-genetic healthcare professionals are invited to complete an online training module about genetic counseling and genetic testing in patients with mPC. After this training, they identify patients who are eligible for genetic testing and provide pre-test counseling. If patients give informed consent, the non-genetic healthcare professional orders germline genetic testing. In most genetics departments the same gene panel will be used, consisting of *BRCA1*, *BRCA2*, *ATM*, *CHEK2* and *PALB2*. Both patient and non-genetic healthcare professional will be informed about the genetic test result by a letter, which is coordinated by the genetics department. If a pathogenic germline variant is found, patients receive an appointment for post-test counseling at the genetics department. If a variant of unknown significance is found, the genetics department will assess whether post-test counseling is indicated.

### Participants

Healthcare professionals from the participating hospitals can participate in the DISCOVER study if they (1) are involved in prostate cancer care, (2) do not have a background in clinical genetics and (3) are willing to complete an online training module that is developed as part of this study.

Patients are eligible for the DISCOVER study if they are diagnosed with metastatic prostate cancer (stage TxNxM1). Prostate cancer patients who have already had a tumor DNA-test will be excluded in this study, as well as men who have had pre-test counseling by a clinical geneticist or genetic counselor. Patients who are unable to understand, speak or write the Dutch language will also be excluded.

**Fig. 2:**
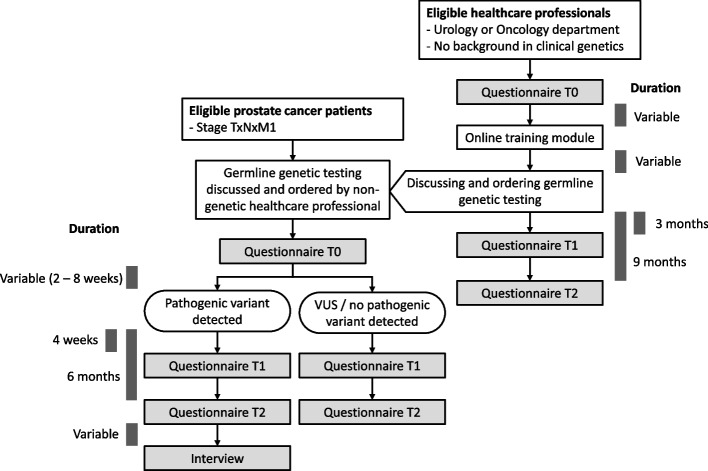
Study protocol for patients and non-genetic healthcare professionals

Figure 2 shows the study protocol in patients and non-genetic healthcare professionals. If the healthcare professional who treats the patient has discussed and ordered germline genetic testing, the first questionnaire will be handed out to the patient. Patients also fill in an informed consent form for the questionnaire study. The second and third questionnaire will be sent to the patient digitally or in print (based on his preference) 4 weeks and 6 months after the patient is informed about the genetic test result. Patients with a pathogenic germline variant will also be invited for an interview. In this interview, the family communication and the consequences for family members will be explored. Non-genetic healthcare professionals who discuss and order germline genetic testing themselves in prostate cancer care will also receive three questionnaires. The first questionnaire will be offered at the start of the online training module. The second and third questionnaire will be sent three and 9 months after completing the online training module.

### Patients

#### Sociodemographic and clinical data

The patients’ age, ethnic background, education level, number and age of children and siblings, treatment history and personal cancer history will be obtained within the first questionnaire. Information about patients’ family history will be gathered through a checklist for healthcare providers. Information about tumor characteristics and TNM stage will be obtained from the Netherlands Cancer Registry and medical records. Germline genetic test results will be collected from the participating departments of Genetics, Urology or Oncology.

The primary research objective is to assess the experience of patients and non-genetic healthcare professionals with the mainstreaming pathway. The measurements for patients are listed in Table 2.

**Table 2:** Study measures in patients

Domain	Measurement	Questionnaire T0	Questionnaire T1		Questionnaire T2	
			**No PV**^**1**^	**PV**^**1**^	**No PV**^**1**^	**PV**^**1**^
Baseline characteristics	Study specific questions	X				
Experience with mainstreaming pathway	Decisional Conflict Scale [50]	X	X	X	X	X
	Decision Regret Scale [51]		X	X	X	X
	Questions adapted from George questionnaire [33]	X	X	X	X	X
	Study specific questions	X	X	X	X	X
Psychosocial impact germline test	Hospital Anxiety and Depression Scale [52]	X	X	X	X	X
	Distress Thermometer [53]	X	X	X	X	X
	Psychosocial Aspects of Hereditary Cancer question 23, 24 [54]	X				
Knowledge	Questions adapted from Claes questionnaire [55]	X	X	X	X	X
Family communication	Informing Relatives Inventory [56]			X		X
	Study specific questions		X		X	
Information regarding pre-test genetic counseling	Study specific questions	X				

^1^pathogenic variant

### Non-genetic healthcare professionals

In the questionnaires for non-genetic healthcare professionals, we will assess (1) their barriers and preferences in the mainstreaming pathway, (2) satisfaction with discussing and ordering of germline genetic testing and with the mainstreaming pathway and (3) experiences with the online training module. In addition, knowledge about genetics in general and genetics in prostate cancer will be assessed. We used adapted questions derived from the questionnaires of Bokkers et al., George et al. and Claes et al. [33, 48, 55].

The secondary research objective is to determine the efficacy of the mainstreaming pathway. First, we will assess the diagnostic yield of germline genetic testing. Second, we will explore the time investment for non-genetic healthcare professionals, which will be assessed with the questionnaires.

### Sample size

We aim to assess the prevalence of pathogenic germline variants with a high accuracy. For that reason, we allow a 95% CI with a maximum width of 3%. With an assumed prevalence of 4%, we need to recruit 800 patients.

### Statistics

Descriptive statistics will be used to characterize the study sample. Baseline and family characteristics of patients with and without pathogenic germline variants will be compared using Student’s t-test and Chi-square test. To assess longitudinal data in our patient cohort we will use generalized linear mixed models. We will also use generalized linear mixed models to assess longitudinal data in our non-genetic healthcare professional cohort.

## Discussion

To the best of our knowledge, our study will be the first to investigate on a larger scale how patients with mPC and non-genetic healthcare professionals experience mainstream genetic testing. It will also be the first to investigate how urologists experience mainstream genetic testing, because other studies mainly focused on medical oncologists, gynecologists and surgeons. In addition, our study will determine the efficacy of the mainstreaming pathway in mPC.

Based on multiple online focus groups and interviews, we determined key topics for our online training module for non-genetic healthcare professionals involved in prostate cancer care. These topics (e.g., the psychosocial and medical consequences of a pathogenic germline variant in breast cancer predisposition gene) were included in the training module. Psychosocial consequences of a pathogenic germline variant could be an increased level of worries, for example about having passed the pathogenic variant to their progeny. In addition, when a pathogenic germline variant is detected, the index patient needs to communicate this with his family, which could be bothersome. In our study we will assess family communication. The medical consequences of a pathogenic germline variant for prostate cancer patients are mainly limited to patients with metastatic castration-resistant prostate cancer who carry a pathogenic variant in the *BRCA1* or *BRCA2* gene. Only in patients with a pathogenic *BRCA* variant PARP inhibitors are used [27]. In addition, prostate cancer patients with a pathogenic germline variant in the *BRCA2* gene have a higher risk of developing metastases and dying from prostate cancer at an early age [8, 57], and they have a higher risk of developing breast cancer [58]. Family members can carry the same pathogenic germline variant and can therefore have a higher risk of developing breast and/or ovarian cancer. Non-genetic healthcare professionals, however, know little about the consequences for family members [59]. Generally, non-genetic healthcare professionals think positively about their own knowledge and about their communication about cancer genetics, but this result was found in non-genetic healthcare professionals who already had experience providing genetics services [59]. Urologists in general have gaps in knowledge about genetic care [60].

Patients in prior studies on mainstream genetic testing in prostate cancer were highly satisfied with mainstream genetic testing [46, 47]. No significant differences were found in distress between patients with or without a pathogenic variant or an uncertain variant [47]. These results should be interpreted with caution because the pathogenic variant group only consisted of three patients. Also, no significant differences were found in distress over time (between 3 weeks and 3 months after their test result), except for the patients with a pathogenic variant [46]. These results were not from prostate cancer patients only, but pooled data from patients with prostate, ovarian and pancreatic cancer. Distress was measured by the MICRA questionnaire in both studies.

All non-genetic healthcare professionals (eight medical oncologists and medical oncology fellows) who were evaluated in Scheinberg’s study were satisfied with the mainstreaming pathway and seven out of eight felt confident to perform pre-test counseling in prostate cancer patients. Urologists and nurse specialists were not included in Scheinberg’s study. In breast and ovarian cancer, nurses or nurse specialists have positive experiences with mainstream genetic testing [34, 36, 38, 44, 48].

To determine the efficacy of the mainstreaming pathway, we will assess the diagnostic yield of pathogenic germline variants in mPC in the Netherlands. The prevalence of pathogenic germline variants is 10% or more in several studies, but in these studies gene panels were used that contained between 13 and 80 genes [4, 6, 7, 10, 12, 14, 15]. Also, not all patient groups were homogeneous because some not only consisted of patients with mPC, but also high-risk, or even low-risk localized prostate cancer patients. A pathogenic germline variant in *BRCA2* was most prevalent in almost all studies, with a prevalence between 1.0 to 8.6% [4, 18]. The prevalence of pathogenic *BRCA2* germline variants in a prior study of 212 Dutch mPC patients was 2.4% [16]. Therefore, the prevalence of *BRCA2* germline variants seems to be low in the Netherlands compared to other countries, but data from larger patient cohorts is needed to confirm this.

A major strength of this study is that multiple centers participate from different regions of the Netherlands. In total, six different genetic centers will participate. In each region, the mainstreaming pathway is adapted to fit best in their daily practice. In addition, we expect to include a large number of patients; substantially more than previous studies. Our study has a few limitations. First, we chose not to set up a comparative study, because we predicted that mainstream genetic testing is increasingly becoming usual care. Therefore, we think that it is not appropriate to recruit such a control group of patients who are counseled by genetic healthcare professionals. Second, because different genetics labs take part in this study and the mainstreaming pathway is adapted to fit best to their daily practice, they all have their own procedure on how non-genetic healthcare professionals can request germline genetic testing. We may be biased in interpreting the efficacy of the mainstreaming pathway, because non-genetic healthcare professionals experience the pathway differently in the participating genetic lab regions. Third, there are no standardized questionnaires for non-genetic healthcare professionals that assess the outcomes of our study. Therefore, we developed study-specific questions and used questions from previous studies [33, 48, 55].

This study will provide more information about the prevalence of pathogenic germline variants in Dutch patients with mPC and will contribute to possible adjustments on germline genetic testing criteria in Dutch guidelines. The results of this study can also be used to further implement mainstream genetic testing in more centers in the Netherlands and abroad, and adjust the current mainstreaming pathway to be more feasible and acceptable.

## Data Availability

Not applicable.

## Abbreviations

AUA: American Urological Association
dpt: department
EAU: European Association of Urology
EMA: European Medicines Agency
FDA: Food and Drug Administration
HCP: healthcare professional
mCPC: metastatic castration-resistant prostate cancer
mPC: metastatic prostate cancer
NCCN: National Comprehensive Cancer Network
PARP: poly (ADP-ribose) polymerase
PV: pathogenic variant
VUS: variant of unknown significance.
